# The Glutaredoxin Gene, *grxB*, Affects Acid Tolerance, Surface Hydrophobicity, Auto-Aggregation, and Biofilm Formation in *Cronobacter sakazakii*

**DOI:** 10.3389/fmicb.2018.00133

**Published:** 2018-02-05

**Authors:** Na Ling, Jumei Zhang, Chengsi Li, Haiyan Zeng, Wenjing He, Yingwang Ye, Qingping Wu

**Affiliations:** ^1^State Key Laboratory of Applied Microbiology Southern China, Guangdong Provincial Key Laboratory of Microbial Culture Collection and Application, Guangdong Open Laboratory of Applied Microbiology, Guangdong Institute of Microbiology, Guangzhou, China; ^2^School of Food Science and Engineering, Hefei University of Technology, Hefei, China

**Keywords:** *Cronobacter sakazakii*, *grxB*, acid stress, biofilm, gene knockout

## Abstract

*Cronobacter* species are foodborne pathogens that can cause neonatal meningitis, necrotizing enterocolitis, and sepsis; they have unusual abilities to survive in environmental stresses such as acid stress. However, the factors involved in acid stress responses and biofilm formation in *Cronobacter* species are poorly understood. In this study, we investigated the role of *grxB* on cellular morphology, acid tolerance, surface hydrophobicity, auto-aggregation (AAg), motility, and biofilm formation in *Cronobacter sakazakii*. The deletion of *grxB* decreased resistance to acid stresses, and notably led to weaker surface hydrophobicity, AAg, and biofilm formation under normal and acid stress conditions, compared with those of the wild type strain; however, motility was unaffected. Therefore, *grxB* appears to contribute to the survival of *C. sakazakii* in acid stresses and biofilm formation. This is the first report to provide valuable evidence for the role of *grxB* in acid stress responses and biofilm formation in *C. sakazakii.*

## Introduction

The *Cronobacter* genus, formerly known as *Enterobacter sakazakii* (Iversen et al., 2007), is a group of opportunistic pathogens that cause rare but life-threatening cases of necrotizing enterocolitis, meningitis, cyst formation, intracerebral infarctions, bacteremia, and sepsis in premature neonates and infants with underlying chronic conditions (NazarowecWhite and Farber, 1997; Bahloul et al., 2017; Jung et al., 2017; Sharma and Melkania, 2017). The International Commission on Microbiological Specification for Foods has ranked *Cronobacter* (*E. sakazakii*) as a “severe hazard for restricted populations, life threatening or substantial chronic sequelae of long duration” (Yan et al., 2012). The presence of *Cronobacter* in powdered infant formula poses high health risks for newborns.

*Cronobacter* spp. exhibits an unusual tolerance to acidic, dry, oxidative, osmotic, and heat stresses compared with other Enterobacteriaceae (Dancer et al., 2009). The ability of *Cronobacter* spp. to withstand adverse acid stresses is one of the key factors responsible for their survival, infection, and pathogenicity. RNA polymerase sigma factor (*rpoS*) and molecular chaperone *hfq* facilitate the defense response of cells of *C. sakazakii* cells to defense against adverse environmental conditions (Álvarez-Ordóñez et al., 2013; Kim et al., 2015). However, the mechanisms underlying the acid stress response of *Cronobacter* spp. are poorly understood.

Biofilm is broadly defined as a microbially triggered adherent matrix-enclosed community attached to a surface or interface (Costerton et al., 1995; Stewart and Franklin, 2008). Biofilms of bacterial pathogens result in the persistence and cross-contamination on contact layers due to the extreme difficulty to eradicate during cleaning, disinfection, and sanitation (Bridier et al., 2015). Ye et al. (2016) used proteomics to compare protein expression during the biofilm and planktonic modes of bacteria to identify unique profiles of protein expression profiles related to biofilms. Recently, researchers also found that quorum sensing signaling molecules acyl homoserine lactones contribute to biofilm formation (Tall et al., 2017).

Glutaredoxin (Grx) systems play a critical role in forming deoxyribonucleotides during DNA synthesis, sensing cellular reduction–oxidation potentials, controlling protein folding, signal transduction, and the regulation of cell processes such as growth, differentiation, and apoptosis (Berndt et al., 2007, 2008; Lillig et al., 2008). Moreover, Grx2, which is encoded by *grxB* and is an important component of Grx systems, contributes up to 80% of total Grx activity in the normal physiological state (Lundberg et al., 2001). Previous research has suggested that the relative expression of *grxB* increased during acetate-induced acid tolerance response in *Escherichia coli*, as assessed by global analysis (Arnold et al., 2001). In addition, the GrxB protein and *grxB* gene were respectively verified to be up-regulated under acidic environment by 2-D electrophoresis and real-time fluorescence quantitative PCR in our laboratory (data not shown). To date, the detailed functions of *grxB* in responses to acid stresses in *C. sakazakii* have not been investigated.

In this study, we compared the morphology, Grx activity, surface hydrophobicity, outer membrane permeability, auto-agglutination, motility, and biofilm formation of *grxB* mutant and parental strains to better understand the role of *grxB* on bacterial phenotypes in *C. sakazakii*, especially exposed to acid stress.

## Materials and Methods

### Strains, Plasmids, and Culture Conditions

All strains and plasmids used in this study are shown in **Table 1**. *C. sakazakii* 1409C1 isolated from ready-to-eat food in China. The primal information of *C. sakazakii* 1409C1 was displayed in Supplementary Table S1. *C. sakazakii* identification was performed by API 20E diagnostic strips (BioMérieux, Marcy-l’Étoile, France), *fusA* sequencing and multilocus sequence typing analysis (Joseph et al., 2012).For genetic manipulation, *E. coli* DH5α and *C. sakazakii* 1409C1 strains were inoculated in Luria-Bertani (LB) medium at 37°C. *E. coli* WM3064 was prepared as described by Jin et al. (2013).

**Table 1:** Strains and plasmids used in this study.

Strain or plasmid	Description	Reference or source
*E. coli* strains	DH5α	Host for cloning	TAKARA
	WM3064	Δ*dapA*, donor strain for conjugation	Jin et al., 2013
	WM3064-G	WM3064 containing PHGM01-Δ*grxB*	This study
*C. sakazakii* strains	1409C1	Wild type	Our laboratory
	Δ*grxB*	Derived from *C. sakazakii* 1409C1	This study
	PHGM01	Ap^r^, Gm^r^ ,Cm^r^ attp, and ccdB	Jin et al., 2013
Plasmids	PHGM01-Δ*grxB*	PHGM01 containing fusion homologous	This study
	pMD19-T	PCR Product Host for cloning	TAKARA

### Construction of Δ*grxB* Mutant

**FIGURE 1:**
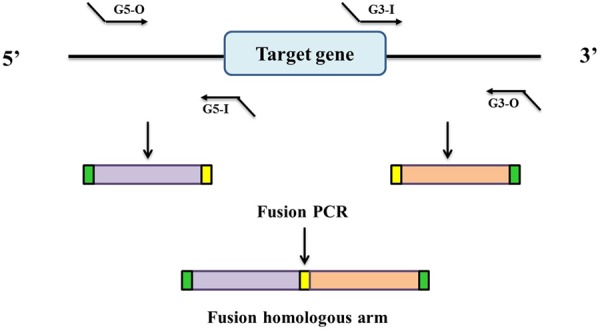
Generation of a fusion PCR product for BP recombination. Outside primers G5-O and G3-O contain attB1 and attB2 sequences, respectively. Inside primers G5-I and G3-I contain linking sequences respectively, which are complementary to each other.

In this study, in-frame deletion strains of *C. sakazakii* were constructed using the high-efficiency bacterial conjugation method (Wan et al., 2015). The primers used for PCR amplification in this study are also presented in **Table 2**. In brief, two fragments flanking the targeted genes were amplified independently by PCR using primers containing the attB sequences (outside primers: G5-O and G3-O) and linking sequences (inside primers: G5-I and G3-I) with genomic DNA as the template; the fragments were then joined together by fusion PCR (**Figure 1**). To fuse the two PCR fragments, 50 μl overlap extension reactions were performed with the following reagents: 1 μl of each of the two PCR fragments, 24 μl PrimeSTAR Max DNA Polymerase mix (Takara Bio, Shiga, Japan), 20 μl ddH_2_O, and 1 μL of each outside primer: G5-O and G3-O (10 μM). The reaction was performed as follows: denaturation for 5 min at 94°C; followed by 30 cycles of 45 s at 94°C, 45 s at 58°C, and 3.0 min at 72°C; and a final extension for 8.0 min at 72°C. DNA obtained after the overlap extension reaction was purified using a PCR purification kit. The resulting fusion homology arm was transformed into plasmid PHGM01. The recombination reaction (5 μl) as follows: 100 ng fusion homology arm, 100 ng PHGM01, 1 μl BP clonase II enzyme mix and TE buffer to 5 μl. Then, recombination was performed for at least 1 h or overnight at 25°C according to the manufacturer’s instructions using the Gateway BP clonase II enzyme mix (Invitrogen, Carlsbad, CA, United States). The mutagenesis vector PHGM01-Δ*grxB* was introduced into *E. coli* WM3064. Thereafter, the correct destination vector for mutagenesis, confirmed by DNA sequencing, was transferred by conjugation into *C. sakazakii*. The deletion mutant was confirmed by sequencing the mutated regions (primers: GLF, GLR, GSF, and GSR).

**Table 2:** Primers of construction of *grxB* mutant.

Identifier	Primer sequence
G5-O	5′-GGGGACAAGTTTGTACAAAAAAGCAGGCTGCTGCTGTTT GTGCTTTGCG-3′
G5-I	5′-GGTCCGGGTTCGCTATCTATTATAACTTC ACGTTTCTCCT-3′
G3-I	5′-ATAGATAGCGAACCCGGACCCGGGC GTCAACTACCCTACC-3′
G3-O	5′-GGGGACCACTTTGTACAAGAAAGCT GGGTCGGTCTTCGCTCAGCACAT-3′
GLF	5′-TGAAGCTGGTGCGTCCTC-3′
GLR	5′-CCCGTAAGTTTCGCTGGTAT-3′
GSF	5′-TGATAGGCGTCGGGACATT-3′
GSR	5′-GTTCGGTGACGGTGTATTGC-3′

### Analysis of Growth of WT and Δ*grxB* Mutant Strains under Acid Stress

The *C. sakazakii* 1409C1 (wild type, WT) and Δ*grxB* strains were inoculated into sterile LB medium overnight at 37°C; thereafter, 1% of each overnight culture was transferred to 5 ml sterile LB at the target pH (3.8, 4, 4.2, 5, 6, and 7) for incubation at 37°C for 66 h with shaking at 180 rpm. Every 30 min, the optical density at 600 nm (OD_600_) was measured and a growth curve was drawn. In addition, the survival situation was determined by counting the number of cells in the control and under sub-lethal acid stress conditions. Aliquots of the cultures (5 ml) were then inoculated into the treatment test tubes containing acidic LB medium (pH 4) and control tubes containing an equivalent volume of neutral LB medium (pH 7), respectively. Samples were incubated overnight on plate count agar to facilitate viable cell counting.

### Morphologic Changes of WT and Δ*grxB* Mutant Strains under Acid Stress

The WT and mutant strains were grown under normal LB and sub-lethal pH LB (pH = 4) at 37°C for 12, 24, 36, and 48 h. Then, pellets were obtained by centrifugation for negative staining with 3% phosphotungstic acid (pH 7.0) for 2 min on carbon-formvar copper grids, following by observation by transmission electron microscopy under an H7500 TEM (Hitachi, Tokyo, Japan) at 80 kVg.

### Biomass Assay of WT and Δ*grxB* Mutant Strains under Acid Stress

WT and Δ*grxB* mutant cells in mid-exponential growth phase were pelleted, and then treated with acid stress (pH 4) for 10, 60, and 180 min. Subsequently, intracellular and extracellular ATP levels were detected as described by Zhang et al. (2011) using a SHG-D luminometer according to the manufacturer’s instructions (Huankai, Guangzhou, China).

### GRX Activity Assay

The WT and mutant strains grown under normal LB and sub-lethal pH LB (pH = 4) were centrifuged and the supernatants were discarded. Culture precipitates were then resuspended in phosphate-buffered saline (PBS). The bacterial suspension was fragmented using a high-pressure cracker to release protein. The protein concentration of the soluble material was determined using a bicinchoninic acid protein assay kit (Beyotime Biotechnology, Jiangsu, China), with bovine serum albumin as a control.

Grx activity was measured as previously described by Li et al. (2005) with a slight modification. The Grx reaction buffer includes 0.5 mM glutathione (GSH), 0.5 mM 2-hydroxyethyl disulfide (HED), 0.5 mM NADPH, 0.5 U/ml GR and 50 mM Tris–HCl. A mixed disulfide containing both HED and GSH was formed within 2 min, and the reaction was started by adding the bacterial crude protein. The rate of the Grx reaction was determined by the decrease in absorbance at 340 nm. One unit of Grx activity was defined as the amount required to oxidize 1 μmol NADPH per minute at 25°C.

### Outer Membrane Permeability of WT and *grxB* Mutant under Acid Stress

Outer membrane permeability of cell was determined as described by Komaniecka et al. (2016) with minor modifications, using a fluorescent hydrophobic probe *N*-phenyl-1-naphthylamine (Sigma-Aldrich, St. Louis, MO, United States). Cells in mid-exponential growth phase were centrifuged and treated by acid medium (pH 4) for 10 min, 1 h, and 3 h. Subsequently, OD_600_ of the cell suspension resuspended with PBS was adjusted to approximately 0.5 and recorded. *N*-phenylnaphthalen-1-amine was added to a final concentration of 40 μM. The fluorescence intensity of the WT and *grxB* mutant suspensions was immediately measured at excitation and emission wavelengths of 340 and 420 nm, respectively.

### Analysis of Cell Surface Hydrophobicity

The cell surface hydrophobicity (CSH) was determined as in a previous report, with minor modifications (Rahman et al., 2008a). Briefly, cells in the mid-exponential growth phase were washed with PBS and bacterial suspensions were adjusted to an OD_600_ of 1.0 (*H*_0_). Bacterial suspension (2 ml) were mixed with 0.4 ml xylene, and then incubated at room temperature for 1 h. The OD_600_ of the aqueous phase was then measured as *H*. The bacterial surface hydrophobicity index (*H*%) was calculated as follows:

$$\begin{array}{c} H\%\;=\;(H_{0}\;-\;H)/H_{0}\;\times \;100\%. \end{array}$$

### Auto-Agglutination Test

Overnight culturing cells were washed twice with PBS, and then resuspended in PBS to an OD_600_ of approximately to 0.6. These initial optical densities were measured and recorded as *A*_0_. The bacteria solutions were stored in tubes at room temperature for 20 h and measured recording as *A*. The auto-aggregation (AAg) percentage (Rahman et al., 2008a) of the bacterial cells was calculated as follows:

$$\begin{array}{c} \mathrm{AAg}\%\;=\;(A_{0}\;-\;A)/A\;\times \;100\%. \end{array}$$

### Motility Assay

Motility assay was performed as previously described, with minor modifications (Rashid and Kornberg, 2000). To assess swimming motility, WT and Δ*grxB* strains were grown to mid-exponential phase, then single colonies were picked onto soft agar motility plates (LB containing 0.3% agar) and incubated at 30°C for 12–14 h. To assess swarming motility, cells were inoculated onto swarming plates (0.5% agar) with a sterile toothpick and observed after 24 h. In addition, cells of WT and *grxB* mutant under different pH conditions (pH 4 and 7) were also assessed for motility.

### Detection of Biofilm

The biofilm-forming abilities of WT and Δ*grxB* mutant isolates were initially determined using crystal violet staining (CVS). *C. sakazakii* isolates were inoculated in LB broth (Huankai) and cultured overnight at 37°C. Cells were subsequently resuspended in LB broth at an OD_600_ of approximately 0.6 and then added to 200 μl LB in 96-cell plates at a dilution of 1:100. The plates were incubated statically at 37°C for 24, 48, 72, and 96 h. The 96-cell plates were rinsed three times with PBS and the adherent cells were stained with 1% crystal violet for 1 h. After rinsing three times with deionized water, the crystal violet was liberated by acetic acid (33%, v/v) following 30 min incubation. The OD_590_ values of each well were measured and recorded.

Overnight cultures with 1% (v/v) were transferred to fresh LB medium with a cell climbing slice (Wohong, Shanghai, China) at 37°C for 24, 48, 72, and 96 h. Subsequently, cells immobilized on the cell climbing slice were examined with an S-3000N scanning electron microscope (Hitachi, Tokyo, Japan) and a confocal laser scanning microscope (Zeiss, Berlin, Germany). Samples were prepared for scanning electron microscopy (SEM) by fixation in 3% glutaraldehyde at 4 °C for 5 h, then dehydration in ethanol followed by tertiary butanol. Dehydrated samples were dried with a CO_2_-critical point dryer, coated with gold, and imaged by SEM at 20 kV.

To better visualize the architecture of the biofilms, biofilms were stained with the LIVE/DEAD BacLight Bacterial Viability Kit (Lot number: L-7012, Molecular Probes, Invitrogen) and observed by confocal laser scanning microscopy (CLSM). In BacLight, propidium iodide and Syto9 were added to stain nucleic acids (Musken et al., 2010). Assessments by SEM and CLSM were performed as described above. The structural parameters of the biofilm (biomass, average thickness, roughness coefficient, and surface-to-volume ratio) were analyzed using COMSTAT program (Heydorn et al., 2000). The maturation stage of biofilm formation was also evaluated in WT and Δ*grxB* strains exposed to low pH medium (pH 4) by CVS, SEM, and CLSM.

## Results

### Comparison of Tolerances to Acid Stress

**FIGURE 2:**
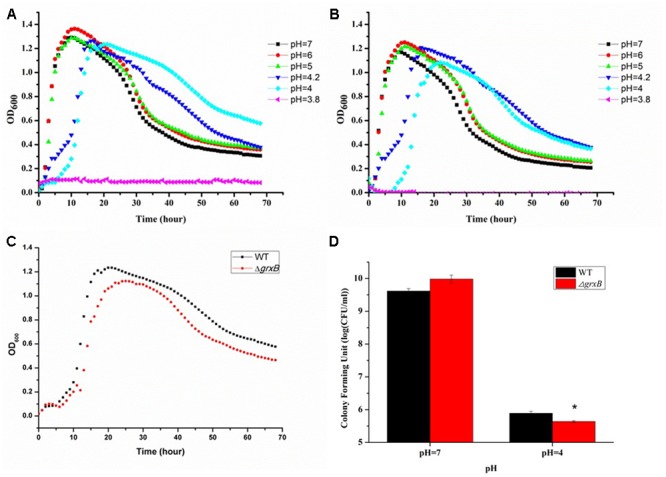
Growth curves and survival of WT and *grxB* mutant under normal conditions and acid stress. **(A)** The growth curves of WT at different pH; **(B)** the growth curves of Δ*grxB* at different pH; **(C)** comparison of growth curve between WT and Δ*grxB* under acid sub-lethal condition (pH = 4); **(D)** survival situation of WT and Δ*grxB* under pH 7 and 4. Values are the mean ± SD of data from three independent experiments. ^∗^*P* < 0.05 vs. WT control cells.

After immersing in culture media with different pH levels (pH 3.8, 4, 4.2, 5, 6, and 7), *C. sakazakii* stains showed a high variability in growth performance (**Figures 2A,B**). The growth curves suggested that acid treatment reduced viability, as it delayed the growth cycles of the parental strain and *grxB*-deficient mutant strains to differing extents. The cells entered exponential phase upon cultivation in LB medium at pH 5, 6, and 7 after 2 h; however, the time to enter the exponential phase was approximately 4 h at pH 4.2 and 6 h at pH 4.0. Approximately identical growth rates were observed in both strains, suggesting that the absence of *grxB* did not affect the initial growth rates of *C. sakazakii.* Furthermore, pH 4.0 was the threshold for this species in laboratory media. The viability of the mutant strain was slightly lower than that of the WT in the sub-lethal acidic conditions (**Figures 2C,D**).

### Evaluation of Biomass under Acid Stress

**FIGURE 3:**
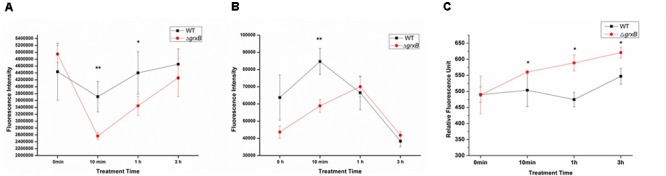
ATP releases of WT and *grxB* mutant under acid stress and outer membrane permeability under acid stress. **(A)** Supernatant ATP; **(B)** pellet ATP; **(C)** outer membrane permeability. Values are the mean ± SD of data from three independent experiments. ^∗^*P* < 0.05 vs. WT control cells; ^∗∗^*P* < 0.01 vs. WT control cells.

ATP, which serves as an indicator for the energy status of cell, was independently measured following different treatment time under the sub-lethal acid stress (**Figure 3**). When the cells were challenged with HCl for 10 min, the intracellular ATP levels of both strains significantly declined and extracellular ATP increased, compared with levels in control cell cultures. In addition, the fluorescence intensity of Δ*grxB* was lower than that of WT (*P* < 0.01) and Δ*grxB* produced less intracellular ATP (*P* < 0.01) at this time. The levels of ATP in the supernatants of the knockout strain also increased and surpassed those of the WT strain, in which extracellular ATP gradually diminished when cells were treated with acid for 1 h. After treatment for 3 h, the intracellular and extracellular ATP levels of both strains almost returned to near their initial levels. With on-going acid treatment over time, both strains tended to have increased permeability; the outer membrane permeability of Δ*grxB* was substantially higher than that of the parental strain at all time points after treatment (**Figure 3C**).

### Cell Behavior under Acid Stress

**FIGURE 4:**
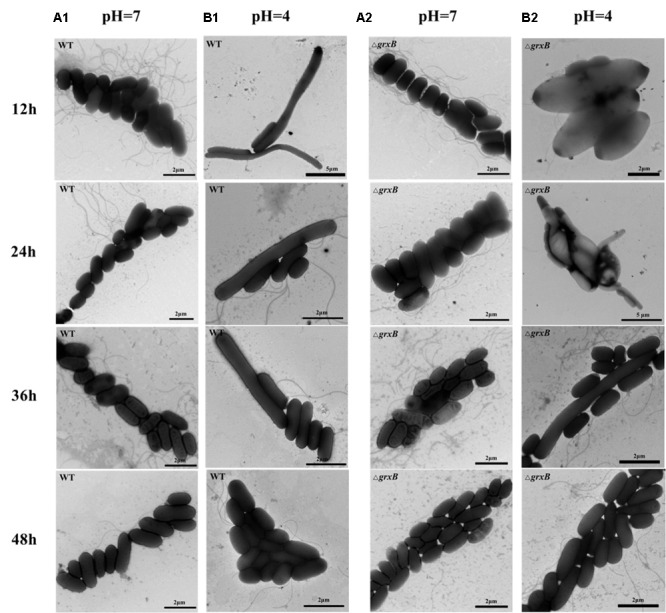
Observation of cell morphology of WT and Δ*grxB*. **(A1,A2)** TEM photographs of WT and Δ*grxB* cultured under pH 7; **(B1,B2)** TEM photographs of WT and Δ*grxB* cultured under pH 4.

To observe the acid adaptation process for an extended period, cell morphologies were respectively detected at pH 7 and 4 using TEM. As shown in **Figure 4**, the acid-treated cells underwent acid adaptation process which WT and Δ*grxB* cells initially underwent morphological distortions, but returned to their normal shapes over time. When the cells were cultured at pH 4 for 12 h, the shapes in both strains changed from slender rhabditiform to thick claviform and the flagella were small; this differed from their morphologies under normal culture conditions. In particular, bulge deformations with intermediate rough and fusiform characteristics similar to a rugby ball were observed in most Δ*grxB* cells. WT and Δ*grxB* strains cultivated in acid medium for 24 and 36 h gradually regained their normal morphologies. Furthermore, no variations were observed in these two strains after culturing in medium at pH 4 for 48 h.

### GRX Activity Assay

The Δ*grxB* strains possessed the diminished Grx activity compared to that of WT (*P* < 0.05), and the loss of Grx activity of Δ*grxB* was consistent under both treatments (**Table 3**). Grx activities of Δ*grxB* were 8.79 and 9.35 mU/g; the WT activities were 11.32 and 11.48 mU/g under normal and low acid conditions, respectively. The values indicate that the deficiency of *grxB* affect the Grx activity to a certain extent.

**Table 3:** Grx activity of WT and Δ*grxB*.

Treatment	Strains	Grx activity (mU/g)
pH 7	WT	11.32 ± 0.98
pH 7	Δ*grxB*	8.79 ± 0.43^∗^
pH 4	WT	11.48 ± 0.42
pH 4	Δ*grxB*	9.35 ± 0.16^∗∗^

*Values are the mean ± SD of data from three independent experiments.^∗^P < 0.05; ^∗∗^P < 0.01 (ΔgrxB vs. WT)*.

### Cell Surface Hydrophobicity, Auto-Agglutination Ability, and Motility

**FIGURE 5:**
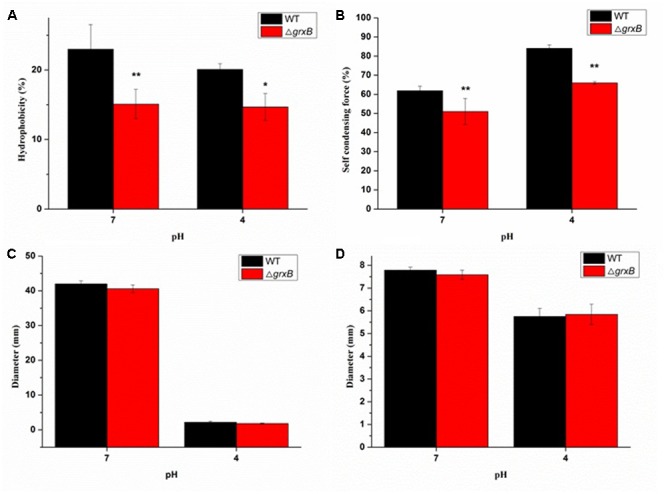
Comparison of biological characterization between WT and *grxB* mutant. **(A)** Cell surface hydrophobicity; **(B)** self-condensing force between WT and Δ*grxB*; **(C)** swimming motility; **(D)** swarming motility. Values are the mean ± SD of data from three independent experiments. ^∗^*P* < 0.05 vs. WT control cells; ^∗∗^*P* < 0.01 vs. WT control cells.

More Δ*grxB* cells remained in the aqueous phase mixed with xylene solution (*P* < 0.01) compared with WT cells, demonstrating that CSH was noticeably reduced after the knockout of *grxB* (**Figure 5A**). Similarly, a significant reduction in CSH of Δ*grxB* was still displayed after acid treatment.

The auto-agglutination assay suggested that WT cells prominently precipitated faster than Δ*grxB* cells in PBS at 25°C for 20 h, and the same phenomenon was also observed when both WT and Δ*grxB* cells were cultured to exponential phase in solutions at pH 4 (**Figure 5B**).

As shown in **Figures 5C,D**, swimming and swarming motility were observed in neutral and acidic media, with few changes in the motility of cells with a deficiency in *grxB.*

### Biofilm Assay and the Relationship between Acid Stress and Biofilm

**FIGURE 6:**
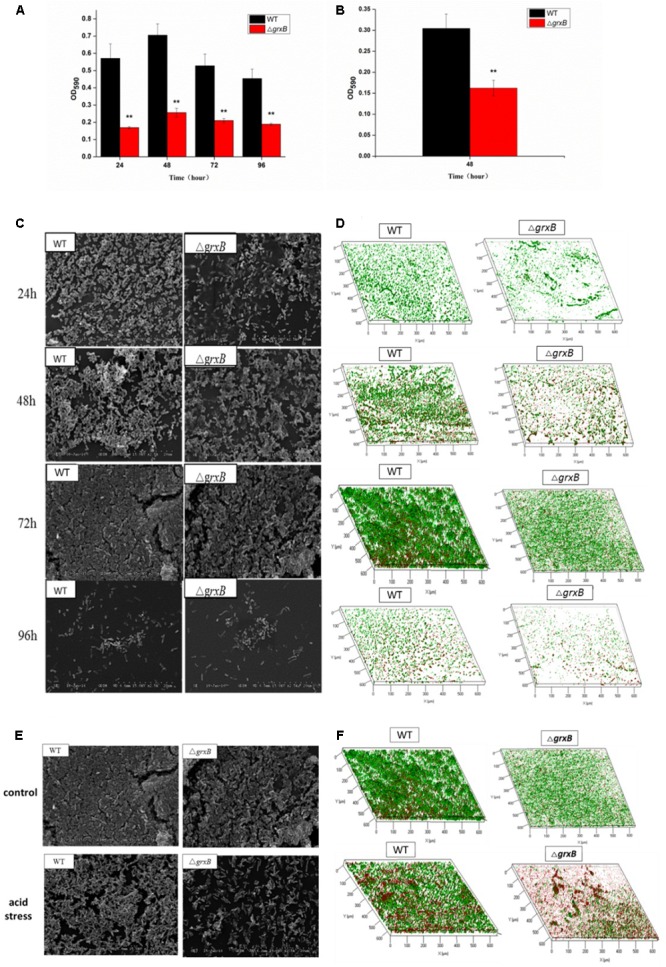
Spatial distribution of biofilms of WT and Δ*grxB* by CVS, SEM, and CLSM. **(A)** Biofilm formation observed by CVS under normal condition; **(B)** biofilm formation observed by CVS under acid condition for 48 h; **(C)** SEM images (×2500) under normal condition; **(D)** CLSM images under normal condition; **(E)** comparison of biofilms between WT and Δ*grxB* under acid condition using SEM; **(F)** comparison of biofilms between WT and Δ*grxB* under acid condition for 72 h using CLSM.

Discrepancies in the biofilm-forming ability were observed between *C. sakazakii* WT and Δ*grxB* isolates by CVS. As shown in **Figure 6A**, Δ*grxB* isolates showed dramatically attenuated biofilm formation at each growth period. The greatest colonization abilities of both strains occurred after culture for 48 h using CVS analysis. Biofilm formation under favorable condition were observed by SEM and CLSM (**Figures 6C,D**), suggesting that biofilm was not just a layer of the closely arranged cells, but many disparate layers with complex substance which distributed a lot of spaces and channels in its internal structure. Furthermore, the Δ*grxB* strains possessed weaker biofilm-forming ability at all stages of growth. The structural parameters (biomass, average thickness, and roughness coefficient) of the mature biofilms cultured for 72 h are shown in **Table 4**. The biovolume is an estimate of the biomass based on the overall volume of the biofilm; the mean thickness indicates the spatial size of the biofilm; the roughness is an indicator of biofilm heterogeneity. The biomass and average thickness of the biofilm were reduced from 13.72 to 11.71 μm^3^/μm^2^ and from 18.69 to 15.05 μm, respectively (WT vs. Δ*grxB*).

**Table 4:** Biomass, average thickness, roughness coefficient and average spots of biofilms of WT and Δ*grxB*.

Strains	Biomass (μm^3^/μm^2^)	Mean thickness (μm)	Roughness
WT	13.72 ± 1.71	18.69 ± 1.98	0.158 ± 0.01
Δ*grxB*	11.71 ± 2.12	15.05 ± 1.52	0.15 ± 0.04
WT_pH	11.17 ± 1.92	19.18 ± 1.65	0.20 ± 0.01
Δ*grxB*_pH	7.09 ± 0.70^∗^	11.44 ± 0.96^∗∗^	0.33 ± 0.05^∗^

*WT_pH, WT cultivated in pH 4; ΔgrxB_pH: ΔgrxB cultivated in pH 4; values are the mean ± SD of data from three independent experiments. ^∗^P < 0.05; ^∗∗^P < 0.01 (ΔgrxB vs. WT, ΔgrxB_pH vs. WT_pH)*.

As external environment can potentially drive cell dysfunction in biofilms, we also analyzed the role of *grxB* on *C. sakazakii* biofilms under acidic conditions. WT and Δ*grxB* strains exposed to media at pH 4 during the mature biofilm stages were utilized for follow-up acid stress using CVS, SEM, and CLSM, respectively. When exposed to acid stress, biofilms of Δ*grxB* isolates were also markedly inhibited compared with biofilms of the WT strain (**Figure 6B**). Additionally, the cells in the biofilms of the two isolates adopted sparse and distributed structures that gathered into a mass, especially in the Δ*grxB* strain (**Figure 6E**). Three-dimensional CLSM images revealed relatively low biofilm formation and a large mass of dead cells for the Δ*grxB* cells compared to WT cultures under acidic conditions (**Figure 6F**). COMSTAT analysis indicated significantly less biofilm activity in sub-lethal acid conditions, as the Δ*grxB* biomass was 7.09 μm^3^/μm^2^ with a thickness of 11.44 μm; the WT biomass was 11.17 μm^3^/μm^2^ with a thickness of 19.18 μm.

## Discussion

Of the environmental factors tested, acid stress was most effective depressor in survival of foodborne pathogens. Acidic food preservatives are frequently applicated in food industry to cause sterilization or bacteriostasis. The survival of foodborne pathogens in the gastrointestinal tract is associated with their acid resistance against the low pH of the stomach. Edelson-Mammel et al. (2006) investigated the acid resistance of 12 strains of *Cronobacter*; they found that *Cronobacter* could withstand exposure to a pH as low as 3.5 for at least 5 h via diverse acid tolerance mechanisms. The sequence of *grxB* is of highly conserved among *Cronobacter* species, and it can unequivocally distinguish *Cronobacter* spp. from other bacteria for rapid detection in powdered infant formula (Dong et al., 2013), and *grxB* is mainly regulated by the stress-related regulator ppGpp and σ^s^ in *E. coli* (Potamitou et al., 2002). However, limited data had been available on the functions of *grxB* in *Cronobacter*. In previous studies, GrxB protein and *grxB* gene were respectively verified to be up-regulated under acidic environment by 2-D electrophoresis and real-time fluorescence quantitative PCR, respectively (data not shown). With the purpose of studying the role of *grxB* in *C. sakazakii* at low pH, *grxB* was knocked out as the most straightforward and effective method to reveal its physiological functions. In our study, we used the gene knockout method of in-frame deletion which could effectively avoid the polar effect caused by other methods such as insertion of resistance gene. The *grxB* mutant was identified using PCR and sequencing.

**FIGURE 7:**
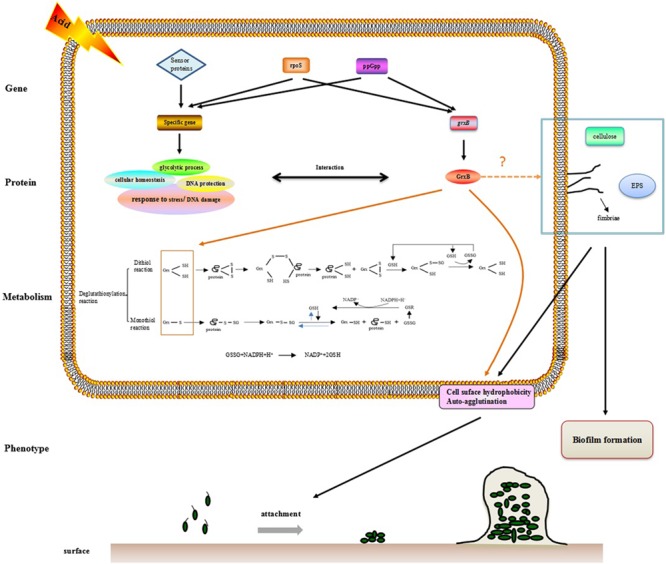
The hypothesis model on mechanism of acid tolerance and biofilm formation in *C. sakazakii*.

The observation that WT and Δ*grxB* bacteria had equivalent growth in normal LB medium suggested that the deletion of *grxB* did not cause a fundamental defect in the initial growth and morphology of *C. sakazakii* (**Figures 2, 4**). However, the structure, substrate utilization rate and intracellular enzyme activity of bacteria can be affected by acid stress, with consequent effects on their growth rate (Hutkins and Nannen, 1993). *C. sakazakii* Δ*grxB* had a lower viability, impaired biomass formation, and greater intracellular ATP leakage in acidic conditions than the WT strain, indicating that *grxB* participates in acid tolerance. Furthermore, the Δ*grxB* strains possessed the diminished Grx activity compared to that of WT. Cytosolic disulfides are kept reduced by the glutaredoxins with NADPH/NADP in the pathway about GSH metabolism (Ströher and Millar, 2012), suggesting that *grxB* expression may contribute to consumption of protons (**Figure 7**). In a previous study, alterations in the cell lengths of *Listeria monocytogenes* and *E. coli* were noted after exposure to high temperatures, acid, and osmotic stress (Jorgensen et al., 1995; Bereksi et al., 2002; Lee et al., 2016). Interestingly, we found that *C. sakazakii* had increasing morphological distortions upon increasing exposure to sub-lethal acid, but returned to their normal shapes over time; this indicated the presence of an acid adaptation process. WT cells developed long filaments, whereas most Δ*grxB* cells adopted the shape of a rugby ball. This dynamic change of aberrant morphology may be associated with an adaptive mechanism to strengthen defense against stress and maintain vital movement by modifying the length, thickness, and size of bacteria.

CSH contributes to hydrophobic interactions between cells and surfaces in aqueous environments (Ellepola et al., 2013). The surface roughness and hydrophobicity of cell membranes determine initial cell adhesion, aggregation, and colony assembly, leading to biofilm development (Myint et al., 2010). Auto-agglutination has also been proposed as an indirect evaluation metric of bacterial absorption ability (Del Re et al., 2000). In this study, CSH and AAg ability noticeably reduced after the knockout of *grxB*, under both normal and acidic growth environment. A good correlation between AAg ability and CSH has previously been described by Rahman et al. (2008b) in *Bifidobacteria*. Attachment of bacteria to a substance is the initial step for biofilm formation where the CSH greatly encourages the adhesion properties in (Habimana et al., 2014; Sahoo et al., 2015). The dramatic alterations in the CSH and aggregation of the mutant *C. sakazakii* suggest a relationship between *grxB* and biofilm formation.

Bacterial biofilms serve as protective microbial barriers against adverse conditions and facilitate adaptation to environmental stresses (Winkelströter et al., 2014). The growth microenvironment affects the formation of *C. sakazakii* biofilms (Ye et al., 2015). Environmental stresses are moderated by the high population densities of biofilms, which act as diffusion barriers. Thus, cells in biofilms generally have more time to react to stress than planktonic and scattered cells in the same conditions (Hall-Stoodley et al., 2004). In this study, biofilm forming ability of Δ*grxB* was relatively weak in normal culture compared to that of WT. Acid profoundly impacted biofilms of WT and mutant strains, and there was a positive correlation between the thickness of biofilms and their biovolumes. In addition, the mutant biofilm had reduced biomass and average thickness in acidified minimal medium. Bacterial flagella mediate motility and are closely involved with biofilm formation (Lemon et al., 2007). The absence of *grxB* had little or no influence on the formation of flagella, as demonstrated by a lack of effect on *C. sakazakii* motility. In *E. coli* (Wood et al., 2006), motility was found to be proportional to the biofilm formation. However, the mutation of vmpA, which is involved the synthesis of c-di-GMP, enhanced the biofilm formation and reduced the motility of *E. coli* K12 (Branchu et al., 2013). Thus, biofilm formation is complex and appears to be species- or strain-dependent. Furthermore, fimbriae, cellulose (Saldana et al., 2009; Hartmann et al., 2010) and extracellular polymeric substances (EPS; Flemming and Wingender, 2010) also play an indispensable role in favoring CSH, auto-agglutination or biofilm formation. The function of *grxB* in biofilm organization is currently unknown; we suspect there may be a link between *grxB*, fimbriae, cellulose, and EPS, all of which lead to biofilm formation.

Finally, we investigated GrxB-interacting proteins in the STRING database of known and predicted protein–protein associations. Interestingly, GrxB resembles GrxA, Dps, TrxB, GapA, PfkA, PfkB, OsmC, YdiZ, and YiaG, which were also related to acid stress and biofilm formation (Choi et al., 2000; Sang et al., 2000; Harrison et al., 2007; Bearson et al., 2009; Zhao and Houry, 2010; Falsetta et al., 2011; Birk et al., 2012). The information on GrxB-interacting partners is displayed in Supplementary Figure S1 and Supplementary Table S2. The associated proteins were mainly enriched for one or more gene ontology term, especially cellular homeostasis and glycolysis. The hypothesis that these biological processes may play important role in acid stress and biofilm formation should to be validated in further experiments (Supplementary Figure S2).

This study provided further evidence that *grxB* in *C. sakazakii* contributes to acid tolerance. Furthermore, *grxB* played a major positive role in CSH, agglutination, and biofilm formation. Additional work on the functions of *grxB* and its interacting proteins will reveal the detailed response mechanism of *C. sakazakii* to environmental stresses and biofilm formation. Understanding the mechanisms of stress tolerance and biofilm formation will play a guiding role in the development of strategies to prevent and treat *C. sakazakii* infection.

## Author Contributions

NL, JZ, QW, and YY conceived the project; NL and YY designed some experiments, analyzed the data, and wrote the article; NL, HZ, CL, and WH performed the experiments; JZ and QW supervised the project; NL complemented the writing.

## Conflict of Interest Statement

The authors declare that the research was conducted in the absence of any commercial or financial relationships that could be construed as a potential conflict of interest.
