# Supplementary material for: The Glutaredoxin Gene, grxB, Affects Acid Tolerance, Surface Hydrophobicity, Auto-Aggregation, and Biofilm Formation in Cronobacter sakazakii
Source: Front Microbiol. 2018 Feb 5;9:133. doi: 10.3389/fmicb.2018.00133 (PMC5807413; doi:10.3389/fmicb.2018.00133)
Supplement: Supplementary file 1 [file Table_1.DOCX]

**The Glutaredoxin Gene, *grxB*, Affects Acid Tolerance, Surface Hydrophobicity, Auto-Aggregation and Biofilm Formation in *Cronobacter sakazakii***

**Na Ling ^1, 2 a^, Jumei Zhang ^1 a^, Chengsi Li ^1^, Haiyan Zeng ^1^, Wenjing He ^1^, Yingwang Ye ^2^ *, Qingping Wu ^1^ ***

*** Correspondence:**Qingping Wu & Yingwang Ye
wuqp203@163.com, [wuqp@gdim.cn](mailto:wuqp@gdim.cn); yeyw04@ mails.gucas.ac.cn

**Supplementary Information contains:**

Supplementary Figures S1, Figures S2, Table S1 and Table S2


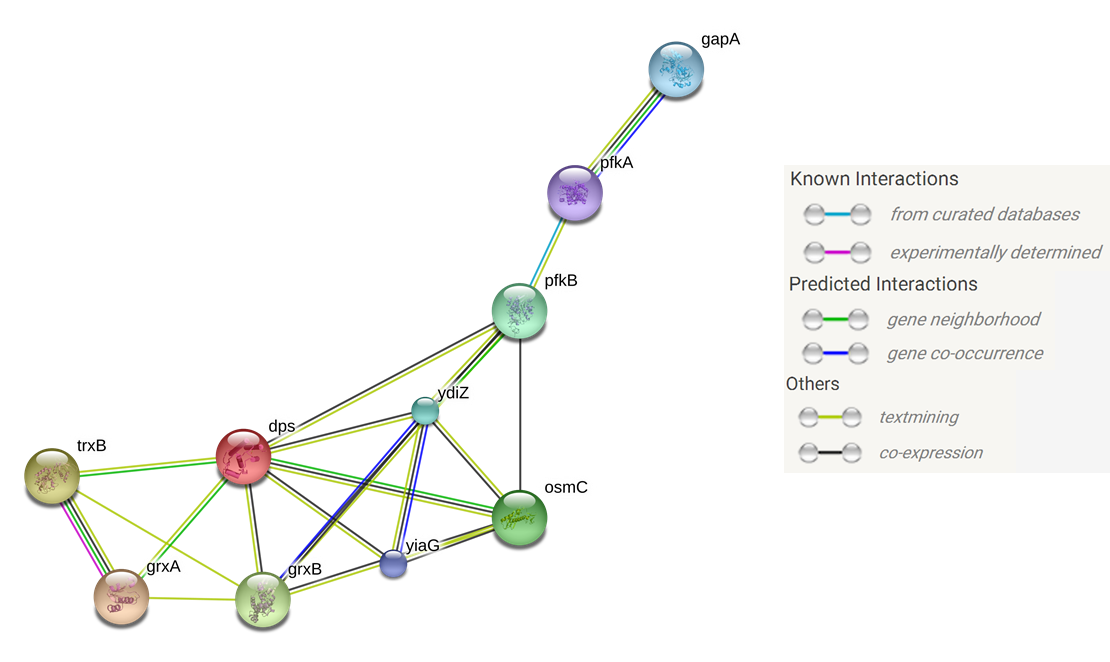


**Figure S1.** Functional protein association network between *grxB* and some proteins related to stress or biofilm. Network nodes represent proteins; small nodes: protein of unknown 3D structure; large nodes: some 3D structure is known or predicted; edges represent protein-protein associations.


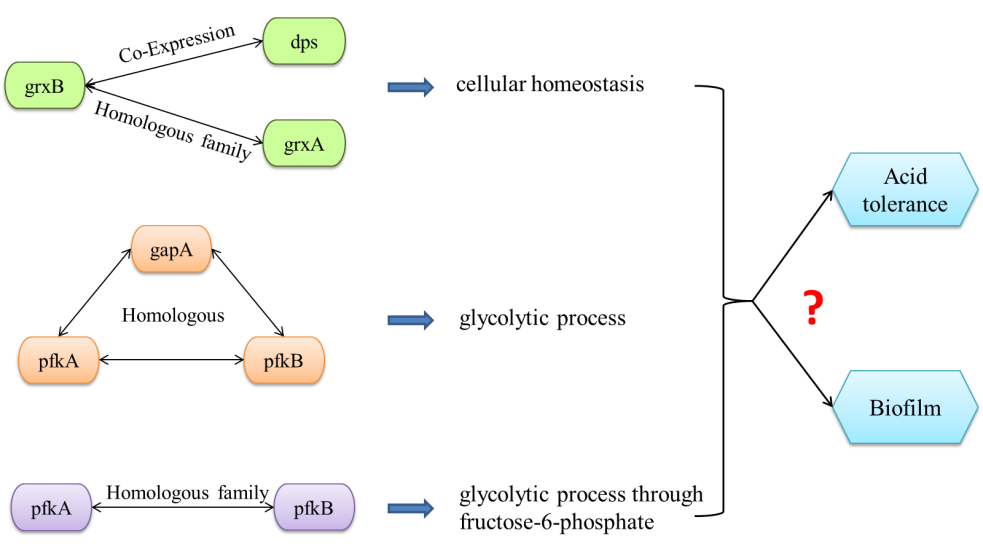


**Figure S2.** The functional context of different genes based on functional Gene Ontology (GO) analysis

**Table S1.** The primal information of *C. sakazakii* 1409C1

| Strain | species | Source | Region | Isolation date (Year) | Serotype | fusA | | ST | |  |
| --- | --- | --- | --- | --- | --- | --- | --- | --- | --- | --- |
| cro1409C1 | *C.sakazakii* | Ready-to-eat food | China | 2013 | O1 | | 67 | | 148 | |

**Table S2.** Information about *grxB* and some proteins interacted with *grxB*

| gene | Annotation | Functional enrichments |
| --- | --- | --- |
| *grxB* | Glutaredoxin 2 (Grx2); Involved in reducing some disulfide bonds in a coupled system with glutathione reductase. Does not act as hydrogen donor for ribonucleotide reductase | cellular homeostasis (GO:0019725) |
| *grxA* | Glutaredoxin 1, redox coenzyme for ribonucleotide reductase (RNR1a); The disulfide bond functions as an electron carrier in the glutathione-dependent synthesis of deoxyribonucleotides by the enzyme ribonucleotide reductase. In addition, it is also involved in reducing some disulfide bonds in a coupled system with glutathione reductase | cellular homeostasis ( GO:0019725) |
| *trxB* | Thioredoxin reductase, FAD/NAD(P)-binding |  |
| *dps* | Fe-binding and storage protein; During stationary phase, binds the chromosome non- specifically, forming a highly ordered and stable dps-DNA co- crystal within which chromosomal DNA is condensed and protected from diverse damages. | cellular homeostasis (GO:0019725) |
| *gapA* | Glyceraldehyde-3-phosphate dehydrogenase A; Catalyzes the oxidative phosphorylation of glyceraldehyde 3-phosphate (G3P) to 1,3-bisphosphoglycerate (BPG) using the cofactor NAD. The first reaction step involves the formation of a hemiacetal intermediate between G3P and a cysteine residue, and this hemiacetal intermediate is then oxidized to a thioester, with concomitant reduction of NAD to NADH. The reduced NADH is then exchanged with the second NAD, and the thioester is attacked by a nucleophilic inorganic phosphate to produce BPG | glycolytic process (GO:0006096) |
| *pfkA* | 6-phosphofructokinase I; Catalyzes the phosphorylation of D-fructose 6-phosphate to fructose 1,6-bisphosphate by ATP, the first committing step of glycolysis | glycolytic process (GO:0006096)  glycolytic process through fructose-6-phosphate (GO:0061615) |
| *pfkB* | 6-phosphofructokinase II; Catalyzes the phosphorylation of D-fructose 6-phosphate to fructose 1,6-bisphosphate by ATP, the first committing step of glycolysis | glycolytic process (GO:0006096)  glycolytic process through fructose-6-phosphate (GO:0061615) |
| *osmC* | Lipoyl-dependent Cys-based peroxidase, hydroperoxide resistance; Preferentially metabolizes organic hydroperoxides over inorganic hydrogen peroxide |  |
| *yiaG* | HTH_CROC1 family putative transcriptional regulator |  |
| *ydiZ* | Predicted protein |  |
