# Supplementary figures and images for: The Glutaredoxin Gene, grxB, Affects Acid Tolerance, Surface Hydrophobicity, Auto-Aggregation, and Biofilm Formation in Cronobacter sakazakii
Source: Front Microbiol. 2018 Feb 5;9:133. doi: 10.3389/fmicb.2018.00133 (PMC5807413; doi:10.3389/fmicb.2018.00133)

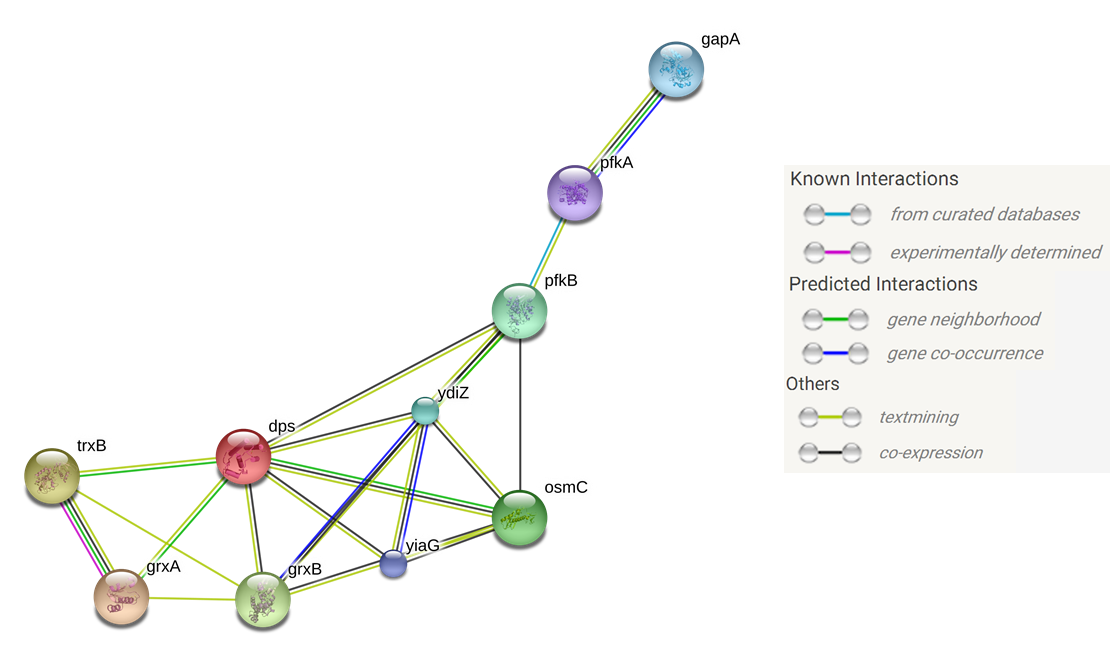

Supplement: Supplementary file 2 [file Image_1.TIF]

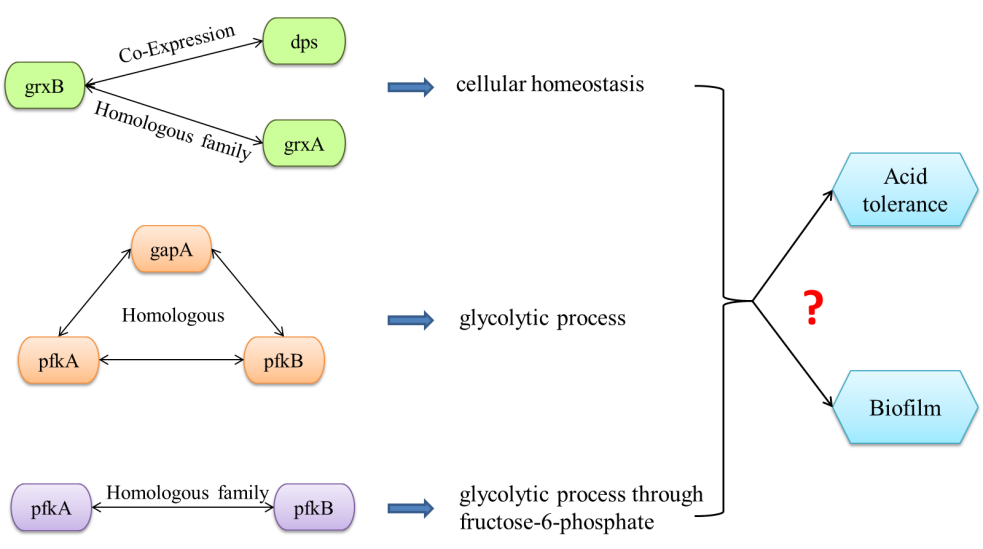

Supplement: Supplementary file 3 [file Image_2.TIF]
